# 高效液相色谱-蒸发光散射检测法同时测定口腔护理产品中10种季铵盐类杀菌剂

**DOI:** 10.3724/SP.J.1123.2023.10013

**Published:** 2024-08-08

**Authors:** Juan TANG, Youchao DING, Xiaoqing FEI, Bin WU, Zhijuan QIAN, Shandan CHEN, Bai LI

**Affiliations:** 1.南京海关工业产品检测中心, 江苏 南京 210019; 1. Industrial Products Testing Center, Nanjing Customs, Nanjing 210019, China; 2.南京海关动植物与食品检测中心, 江苏 南京 210019; 2. Animal, Plant and Food Inspection Center, Nanjing Customs, Nanjing 210019, China; 3.南京海关轻工产品与儿童用品检测中心, 江苏 扬州 225009; 3. Light Industrial Products and Children’s Products Inspection Center, Nanjing Customs, Yangzhou 225009, China; 4.南京金检检验有限公司, 江苏 南京 210019; 4. Nanjing Jinjian Inspection Co., Ltd., Nanjing 210019, China

**Keywords:** 高效液相色谱, 蒸发光散射检测, 季铵盐, 杀菌剂, 口腔护理产品, high performance liquid chromatography (HPLC), evaporative light-scattering detection (ELSD), quaternary ammonium salt, bactericides, oral care products

## Abstract

建立了同时测定口腔护理产品中10种季铵盐类杀菌剂的高效液相色谱-蒸发光散射(HPLC-ELSD)检测方法。在采用无水硫酸钠脱水后的膏体样品和粉末样品中加入乙醇,常温超声提取;液体样品用乙醇稀释。采用Acclaim Surfactant色谱柱(150 mm×4.6 mm, 5 μm)进行分离,以50 mmol/L乙酸铵缓冲溶液(pH=5.5)和乙腈为流动相进行梯度洗脱,外标法定量。结果表明,10种目标化合物在25 min内完成色谱分离,检出限(信噪比(*S/N*)=3))和定量限(*S/N*=10)分别为1.42~3.31 mg/L和4.25~9.94 mg/L。十八烷基二甲基苄基氯化铵和二十二烷基三甲基氯化铵在10~200 mg/L范围内线性关系良好,其余目标物在5~100 mg/L范围内线性关系良好,10种季铵盐类杀菌剂的相关系数(*R*^2^)均不小于0.9992。以阴性样品牙膏(膏体)、漱口水(液体)和洁牙粉(粉末)为样品基质,在不同添加水平下,10种季铵盐类杀菌剂的平均回收率为87.9%~103.1%,相对标准偏差(RSD, *n*=6)均不大于5.5%。本方法在液相色谱分离时采用表面活性剂色谱柱,目标化合物均获得较好保留,同时采用蒸发光散射检测器,部分无紫外吸收的目标化合物也能得到较高响应。通过添加适量无水硫酸钠去除膏体样品中的水分,有利于样品前处理和保护色谱柱。本方法精密度好,准确度高,适用于各种口腔护理产品中季铵盐类杀菌剂的测定,为口腔护理产品的质量安全监控提供了参考。采用本方法对109个常用口腔护理产品进行测定,十六烷基二甲基苄基氯化铵和十八烷基二甲基苄基氯化铵的检出率较高,1个牙膏样品中十八烷基二甲基苄基氯化铵的含量超出法规要求,测定值为1511 mg/kg。

## Abstract (en)

Quaternary ammonium salt bactericides are broad-spectrum bactericides often used in oral care products because of their high antibacterial efficacy, strong penetration, and low toxicity. However, the excessive use of quaternary ammonium salt bactericides may cause contact dermatitis, scalding poisoning, and even death. Existing methods to determine quaternary ammonium salt bactericides are unable to meet current requirements owing to the lack of determination components. Therefore, establishing a simple and accurate method for the simultaneous detection of more quaternary ammonium salt bactericides is necessary.

In this study, a method that couples sample pretreatment with high performance liquid chromatography-evaporative light-scattering detection (HPLC-ELSD) was developed for the simultaneous determination of quaternary ammonium salt bactericides in oral care products, including dodecyltrimethylammonium chloride, dodecyldimethylbenzylammonium chloride, benzethonium chloride, tetradecyl trimethyl ammonium chloride, tetradecyldimethylbenzylammonium chloride, *N*-hexadecyltrimethylammonium chloride, benzyldimethylhexadecylammonium chloride, trimethylstearylammonium chloride, stearyldimethylbenzylammonium chloride, and docosyltrimethylammonium chloride. Some of these bactericides do not absorb ultraviolet light, so a universal evaporative light-scattering detector was used owing to testing cost and stability concerns. The paste samples contained thickening agents, which are highly soluble in water but insoluble in organic solvents; these agents can seriously affect the results of sample pretreatment and damage the chromatographic column. Hence, sample dehydration was necessary. In this study, four dehydration methods were compared. Anhydrous sodium sulfate (Na_2_SO_4_) was selected, and the amount of Na_2_SO_4_ was optimized. Based on the solubility of the 10 target compounds and extraction efficiency, three extraction solvents were compared, and ethanol was selected. Ultrasonic extraction was the primary extraction process used in this study. The effects of different ultrasonication times, temperatures, and powers on the extraction recoveries were also investigated. Ultimately, the optimized conditions were as follows: extraction of the dehydrated paste and powder samples using ethanol at room temperature (25 ℃) for 20 min under 100 W ultrasound power, and dilution of the liquid sample with ethanol.

After extraction, the samples were separated on an Acclaim Surfactant column (150 mm×4.6 mm, 5 μm) with 50 mmol/L ammonium acetate aqueous solution (pH=5.5) (A) and acetonitrile (B) as mobile phases. The gradient elution program were as follows: 0-5.0 min, 75%A-35%A, 5.0-15.0 min, 35%A-20%A, 15.0-20.0 min, 20%A, 20.0-21.0 min, 20%A-75%A, 21.0-25.0 min, 75%A. An external standard method was used for quantitative determination. The 10 compounds were analyzed within 25 min. Linear equations, correlation coefficients, and linear ranges were obtained by analyzing a series of mixed standard working solutions. The limits of detection (LODs, *S/N*=3) and quantification (LOQs, *S/N*=10) of the 10 components were determined. Stearyldimethylbenzylammonium chloride and docosyltrimethylammonium chloride showed good linear relationships in the range of 10-200 mg/L, while the other compounds demonstrated good linear relationships in the range of 5-100 mg/L. In all cases, correlation coefficients (*R*^2^) of no less than 0.9992 were obtained. The LODs and LOQs were in the range of 1.42-3.31 mg/L and 4.25-9.94 mg/L, respectively. Ten analytes were spiked in blank matrices, such as toothpaste (paste), mouthwash (liquid), and dentifrice powder (powder) at three levels, and the recoveries and precisions were calculated. The average recoveries were 87.9%-103.1%, and the corresponding relative standard deviations (RSDs) did not exceed 5.5% (*n*=6). The developed method was used to detect 109 oral care products. Benzyldimethylhexadecylammonium chloride and stearyldimethylbenzylammonium chloride revealed high detection rates. Moreover, the amount of stearyldimethylbenzylammonium chloride in one toothpaste sample exceeded regulatory requirements. Given its advantages of good precision and accuracy, the developed method is suitable for the quantitative analysis of the 10 aforementioned compounds in typical oral care products. The study findings can serve as a reference for the quality and safety monitoring of oral care products.

季铵盐类杀菌剂是一种阳离子表面活性剂类的广谱性杀菌剂,具有杀菌效率高、渗透能力强以及毒性低等特点^[1]^。1915年,Jacobs W首次合成出季铵盐类化合物,并发现此类化合物具有一定的杀菌能力;1935年,德国人Domark Gf发现二甲基氯化铵具有较强的杀菌能力,利用其处理军服,大大降低了伤口感染几率;同年,Wetzel R将季铵盐类杀菌剂应用于临床消毒^[2]^。但已有报道表明过量使用季铵盐类杀菌剂会引起变态反应性结膜炎、视力减退、接触性皮炎、灼伤中毒,甚至死亡^[3,4]^。

美国、欧盟、日本等均将口腔护理产品纳入化妆品管理,我国也于2020年将口腔护理产品参照普通化妆品的相关规定进行管理。《化妆品安全技术规范》(2015版)和欧盟化妆品规程(Council Directive 76/768/EEC)中明确规定化妆品中十二烷基二甲基苄基氯化铵、十四烷基二甲基苄基氯化铵、十六烷基二甲基苄基氯化铵和十八烷基二甲基苄基氯化铵总量不得超过0.1%(质量分数);对于驻留类产品,十二烷基三甲基氯化铵、十四烷基三甲基氯化铵、十六烷基三甲基氯化铵、十八烷基三甲基氯化铵和二十二烷基三甲基氯化铵的使用量不大于0.25%(质量分数);对于淋洗类产品,十六烷基三甲基氯化铵和十八烷基三甲基氯化铵的使用量不得超过2.5%(质量分数),二十二烷基三甲基氯化铵的使用量不得大于5.0%(质量分数);苄索氯铵的使用量不得超过0.1%(质量分数)。

目前与季铵盐类杀菌剂检测相关的标准主要有QB/T 5452-2019、GB/T 30931-2014、GB/T 26369-2020、GB/T 40185-2021和GB/T 39873-2021^[5⇓⇓⇓-9]^。上述标准只建立了部分季铵盐类杀菌剂的检测方法,且主要集中于有紫外吸收的化合物。而化妆品规范和欧盟规程需要考察的季铵盐类杀菌剂多达10种,因此目前的检测标准不能满足口腔护理产品归类为化妆品后季铵盐类杀菌剂的检测需求。通过文献调研可知,目前常见的季铵盐类化合物检测方法有液相色谱-质谱法^[9⇓⇓⇓-13]^、气相色谱-质谱法^[14]^、高效液相色谱法^[5⇓⇓-8,15⇓⇓-18]^、毛细管电泳法^[7,19,20]^、分光光度法^[21,22]^、滴定法^[7,23]^等,研究的基质主要有消毒产品、牙膏、纺织品、冷冻饮品和奶粉等。其中质谱检测器推广成本较高,毛细管电泳仪普及率不如高效液相色谱仪高,滴定法和分光光度法干扰较多,且上述文献研究的季铵盐类杀菌剂种类较少。因此迫切需要建立一种同时测定口腔护理产品中10种季铵盐类杀菌剂的方法。

本工作建立了一种可同时测定口腔护理产品中10种季铵盐类杀菌剂的高效液相色谱-蒸发光散射检测(HPLC-ELSD)方法,该方法可满足相关技术规范和欧盟规程的检测需求,为口腔护理产品的品质安全监管提供技术支持,具有较高的应用价值。

## 1 实验部分

### 1.1 仪器、试剂和材料

1260型高效液相色谱仪,配备蒸发光散射检测器和二极管阵列检测器(DAD)(美国Agilent公司); KQ-250DB型数控超声波清洗器(昆山市超声仪器有限公司); arium^®^ pro DI型纯水机(德国赛多利斯公司); FD56型干燥箱(德国BINDER公司); YB-C型真空恒温干燥箱(天津药典标准仪器厂); PL602-L型和ML54型电子天平(感量0.01 g和0.0001 g,梅特勒-托利多上海有限公司)。

乙醇和乙腈(色谱纯,德国Merck公司);乙酸铵(色谱纯,美国Sigma-Aldrich公司);冰乙酸和无水硫酸钠(分析纯,南京化学试剂股份有限公司)。

标准品:十二烷基三甲基氯化铵(CAS No. 112-00-5,纯度≥99%)、十四烷基三甲基氯化铵(CAS No. 4574-04-3, 纯度≥98%)、十六烷基三甲基氯化铵(CAS No. 112-02-7, 纯度≥99%)、十八烷基三甲基氯化铵(CAS No. 112-03-8, 纯度≥98%)、十八烷基二甲基苄基氯化铵(CAS No. 122-19-0, 纯度≥98%)和二十二烷基三甲基氯化铵(CAS No. 17301-53-0, 纯度≥85%)均购于上海麦克林生化科技有限公司;十二烷基二甲基苄基氯化铵(CAS No. 139-07-1,纯度≥98%)和十四烷基二甲基苄基氯化铵(CAS No. 139-08-2, 纯度≥98%)购于德国Dr Ehrenstorfer公司;苄索氯铵(CAS No. 121-54-0, 纯度≥99%)购于美国AccuStandard公司;十六烷基二甲基苄基氯化铵(CAS No. 122-18-9, 纯度≥95%)购于上海安谱实验科技股份有限公司。

### 1.2 标准溶液的配制

标准储备液:分别准确称取一定量的季铵盐类杀菌剂标准品,用乙醇溶解并定容至10 mL,配制成质量浓度均为10 g/L的单一标准储备液。

混合标准工作液:分别移取一定体积上述10种标准储备液,用乙醇配制成混合标准工作液,其中十八烷基二甲基苄基氯化铵和二十二烷基三甲基氯化铵的质量浓度为400 mg/L,其余均为200 mg/L。

### 1.3 样品前处理

#### 1.3.1 膏体样品

称取1.0 g(精确至0.01 g)样品于50 mL离心管中,加入5 g无水硫酸钠搅拌混匀后,加入10 mL乙醇,常温超声提取20 min,以8000 r/min离心3 min后,取部分上清液,过膜后供HPLC-ELSD测定。

#### 1.3.2 液态水基样品

称取1.0 g(精确至0.01 g)样品于10 mL容量瓶中,用乙醇定容至刻度,混匀,取部分溶液,过膜后供HPLC-ELSD测定。

#### 1.3.3 粉末样品

称取1.0 g(精确至0.01 g)样品于50 mL离心管中,加入10 mL乙醇,常温超声提取20 min,以8000 r/min离心3 min后,取部分上清液,过膜后供HPLC-ELSD测定。

### 1.4 分析条件

色谱柱:Acclaim Surfactant(150 mm×4.6 mm, 5 μm,美国Thermo Scientific公司);流动相:A-50 mmol/L乙酸铵缓冲溶液(pH=5.5), B-乙腈;梯度洗脱程序:0~5.0 min, 75%A~35%A; 5.0~15.0 min, 35%A~20%A; 15.0~20.0 min, 20%A; 20.0~21.0 min, 20%A~75%A; 21.0~25.0 min, 75%A。色谱柱温度:30 ℃;进样量10 μL;流动相流速1.0 mL/min;分析时间25 min;蒸发光散射检测器,蒸发温度40 ℃,雾化器温度40 ℃,雾化气体为氮气,载气流速1.5 L/min。

## 2 结果与讨论

### 2.1 检测器的选择

采用DAD,在0~30 min和190~700 nm范围内,对20 mg/L的10种季铵盐类杀菌剂标准溶液作全波长扫描。结果表明,除十二烷基二甲基苄基氯化铵、十四烷基二甲基苄基氯化铵、十六烷基二甲基苄基氯化铵、十八烷基二甲基苄基氯化铵和苄索氯铵外,其余5种待测分析物几乎没有紫外吸收,可见采用DAD不能获得有效响应,这也是常见的检测标准和文献研究中只涉及3~5种季铵盐类杀菌剂的原因。ELSD、示差检测器(RI)和质谱(MS)均是通用型检测器,但RI灵敏度较差,且不适用于梯度洗脱,而MS成本过高不易推广。郑国建等^[24]^采用HPLC-ELSD法测定了消毒剂中的7种季铵盐,解决了无紫外吸收的季铵盐无法使用DAD的问题。因此,本研究选择ELSD作为检测器。

### 2.2 色谱柱的选择

文献中主要采用C18柱^[24,25]^、CN柱^[26⇓-28]^和SCX柱^[29]^等作为季铵盐类化合物的分离色谱柱,本研究涉及的多种季铵盐类化合物也可用作阳离子表面活性剂,而Surfactant柱是专门用于分离阴离子、阳离子、非离子和两性表面活性剂的色谱柱。因此,本实验以100 mmol/L乙酸铵(pH=5.5)和乙腈作为流动相,分别比较了C18(150 mm×4.6 mm, 5 μm)、CN(150 mm×4.6 mm, 5 μm)、SCX(150 mm×4.6 mm, 5 μm)和Surfactant(150 mm×4.6 mm, 5 μm) 4种色谱柱对10种季铵盐类杀菌剂的分离效果。经实验可知,Surfactant柱对10种季铵盐类杀菌剂的分离效果最佳,C18柱分离效果最差。文献[3]中指出,在选用C18柱作为季铵盐类化合物的分离色谱柱时,需在流动相中添加庚烷磺酸钠和三乙胺才能得到较好的分离效果,但只有挥发性流动相添加剂可用于ELSD,庚烷磺酸钠不符合使用要求;CN柱和SCX柱的分离时间较长且分离效果不理想,因此,本研究采用Surfactant柱。

### 2.3 流动相的优化

甲醇和乙腈为液相色谱分析时最常使用的有机流动相,但实验表明,甲醇会导致Surfactant柱的背景压力过高,而使用任何比例的乙腈,色谱柱的背景压力均适中,因此本研究选择乙腈作为有机流动相。

流动相中挥发性盐的离子浓度是影响Surfactant柱分离效果的重要因素之一,实验比较了不同浓度(0~100 mmol/L)的乙酸铵缓冲溶液对10种季铵盐类杀菌剂色谱分离的影响。经试验可知,待测分析物的保留时间随着离子浓度的增加而增加,当乙酸铵的浓度为50 mmol/L时,各分析物均获得较理想的保留时间和分离效果。

pH值是影响Surfactant柱分离效果的另一个重要因素,Surfactant柱的最佳使用pH值范围是3.0~6.5, ELSD的使用说明书要求使用乙酸铵缓冲盐时pH值范围是3.8~5.8和8.2~10.2。结合Surfactant柱和ELSD的使用要求,实验比较了pH值为4.0、4.5、5.0、5.5和5.8的50 mmol/L乙酸铵缓冲溶液(乙酸调节pH)对10种季铵盐类杀菌剂的色谱分离效果。经试验可知,随着pH值的增大,各分析物的保留时间均有所增加,其中十二烷基二甲基苄基氯化铵、苄索氯铵和十四烷基三甲基氯化铵之间的分离度以及十八烷基三甲基氯化铵和十八烷基二甲基苄基氯化铵之间的分离度增加明显,当乙酸铵溶液的pH值为5.5时,10种待测化合物获得最佳的分离效果。优化的色谱条件下10种季铵盐类杀菌剂的液相色谱图见图1。

**图1:**
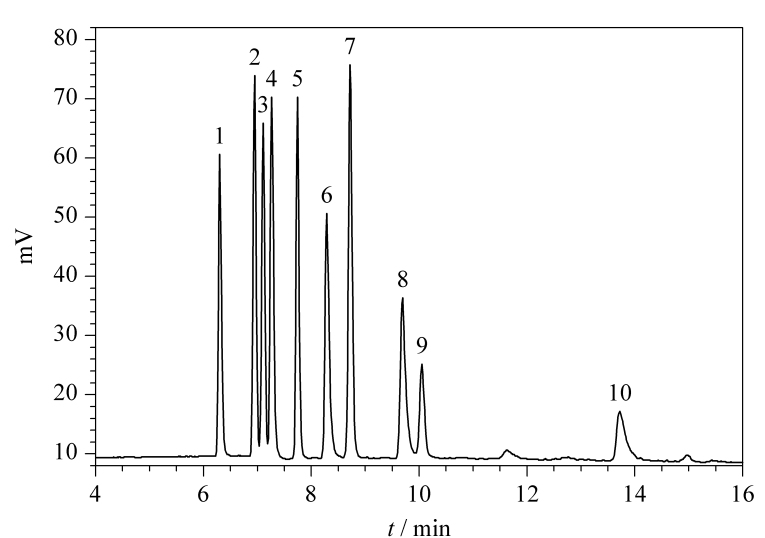
10种目标分析物的色谱图

3号峰(BZC)与4号峰(TTAC)的分离度为1.43,未达1.5。为了进一步验证定量数据的准确性,本研究选取一个空白样本,添加BZC和TTAC标准样品,加标量均为100 mg/kg,然后进行分析。分别采用所建立的混标定量标准曲线和BZC、TTAC的单标定量标准曲线对BZC和TTAC进行定量分析。3号峰BZC采用混标定量和单标定量的结果分别是97.38 mg/kg和100.21 mg/kg,两个结果的差值占平均值的2.86%, 4号峰TTAC的两种结果分别是98.77 mg/kg和101.88 mg/kg,两个结果的差值占平均值的3.10%。因此,虽然3号峰与4号峰的分离度未达到1.5,但对最终的定量结果影响较小。

### 2.4 前处理条件的优化

#### 2.4.1 膏体样品脱水优化

膏体样品含有一些易溶于水不易溶于有机试剂的增稠剂,试验表明,如直接使用有机试剂提取,部分增稠剂溶于有机试剂与水的混合物中,获得的提取溶液过滤较困难,长时间进样也会堵塞色谱柱,缩短色谱柱使用寿命。因此需对膏体样品进行脱水处理。常用的脱水方法有普通烘箱75 ℃或105 ℃烘干、真空干燥75 ℃烘干和无水硫酸钠脱水;采用普通烘箱烘干和真空干燥烘干时膏体样品称取后直接置于50 mL玻璃烧杯中,采用无水硫酸钠脱水时膏体样品称取后直接置于50 mL离心管中。经试验可知,采用普通烘箱烘干和真空干燥烘干这3种条件会导致膏体样品结块,而采用无水硫酸钠进行脱水时,样品呈沙状,不会结块,且增加了样品与提取溶剂的接触面,更有利于目标物的提取。

实验对无水硫酸钠的用量进行了优化。以1 g膏体样品为研究对象,分别加入1、2、3、4、5、6和7 g无水硫酸钠,搅拌均匀后考察样品状态。本试验选取各品牌共28个膏体样品进行考察,试验结果表明,5 g无水硫酸钠与其中26个膏体样品搅拌均匀后呈沙状。少数不能呈沙状的样品,可适当增加无水硫酸钠的添加量。

膏体样品经过脱水处理后,可避免大部分的增稠剂进入到提取液中,样品提取液经离心和过膜处理也可去除大部分进入提取液的增稠剂或杂质。为了进一步验证乙醇作为提取溶剂时提取出的增稠剂对色谱柱的影响程度,补充试验如下:将1个膏体样品提取液每天进样50次,周期为1周,色谱柱柱压从7.2 MPa增加至7.6 MPa,变化较小,说明已去除大部分增稠剂。

#### 2.4.2 提取溶剂的选择

本研究涉及的季铵盐类化合物易溶于甲醇、乙醇或丙酮,以阴性牙膏样品和洁牙粉样品为研究基质,以添加回收的方式考察了3种超声提取溶剂对待测分析物提取效率的影响,结果如图2所示。以乙醇作为提取溶剂时,牙膏和洁牙粉中待测化合物的回收率为71.9%~82.6%和72.9%~86.3%,而采用甲醇和丙酮为提取溶剂时,待测化合物的回收率普遍低于以乙醇为提取溶剂时的回收率。考虑到乙腈破坏胶束的能力较强,对于季铵盐类化合物的提取比较有利,本研究又对乙醇、乙腈、酸化乙腈3种提取溶剂的提取效果进行了比较,结果表明乙醇的提取效果略高于其他两种提取溶剂,基于环保和实验成本的考虑,本研究最终选择乙醇作为提取溶剂。

**图2:**
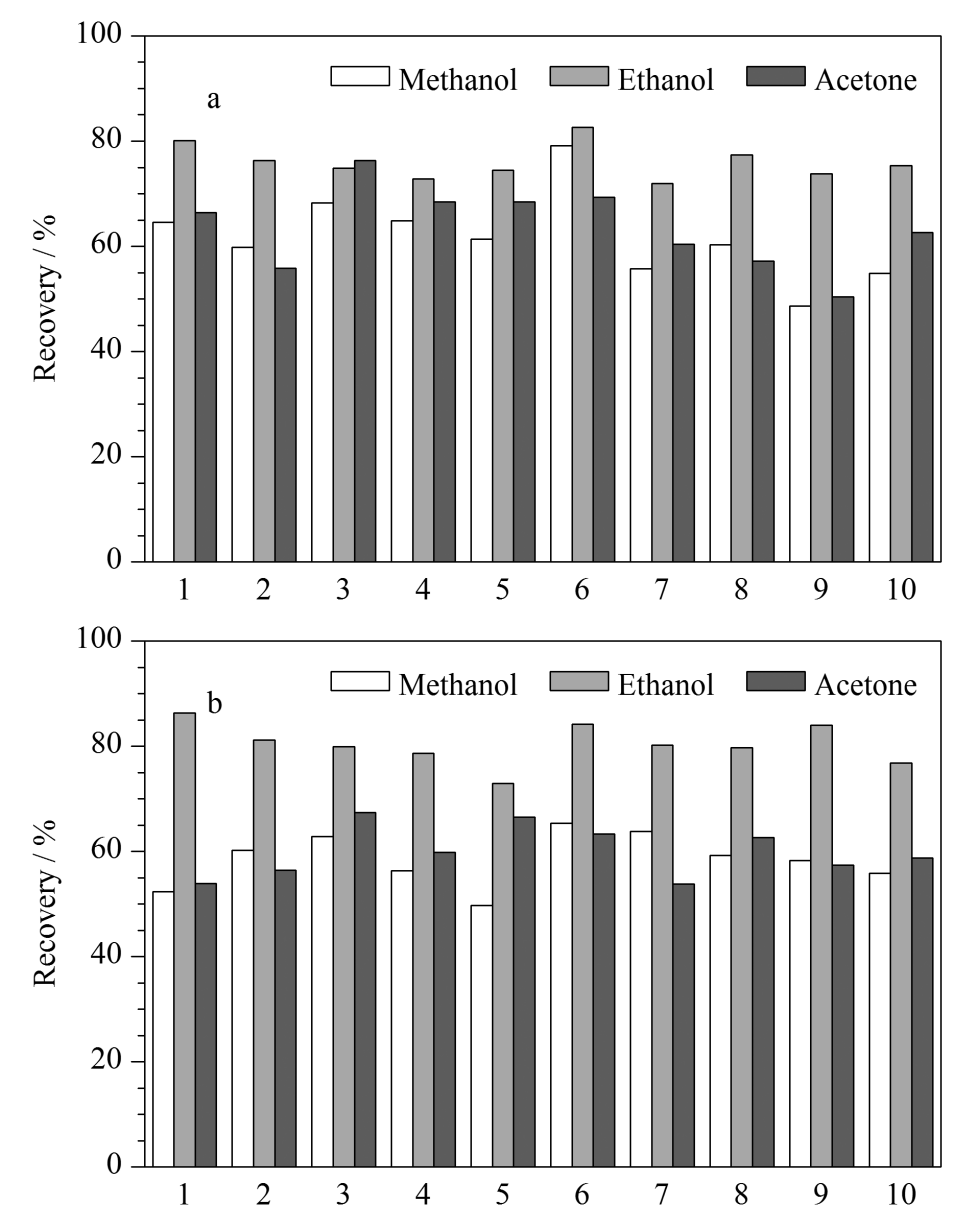
不同提取溶剂对10种目标分析物提取效率的影响

#### 2.4.3 提取条件的优化

本研究采用超声提取作为样品提取的方式,影响超声提取效率的关键因素主要有超声时间、超声温度和超声功率。经试验可知,随着超声温度或超声功率的提高,虽有利于提高待测化合物的提取率,但温度超过室温(25 ℃),超声功率大于100 W时,膏体样品和粉末样品中杂质的提取量也随之变高,含有增稠剂的膏体样品更是变得浑浊难以过滤。经优化,本研究选择25 ℃下超声,超声功率为100 W。

实验以阴性牙膏样品和洁牙粉样品为研究基质,以添加回收的方式分别考察了不同超声时间(0、10、20、30、40 min)对分析结果的影响,实验结果见图3。随着超声时间的延长,10种待测分析物的提取率也随之提高,当超声时间为20 min时,各分析物的回收率均不小于90.9%,继续延长超声时间,一些化合物的回收率虽然还能小幅提高,但溶解在提取溶液中的杂质也会增加,因此,本研究将超声时间设为20 min。

**图3:**
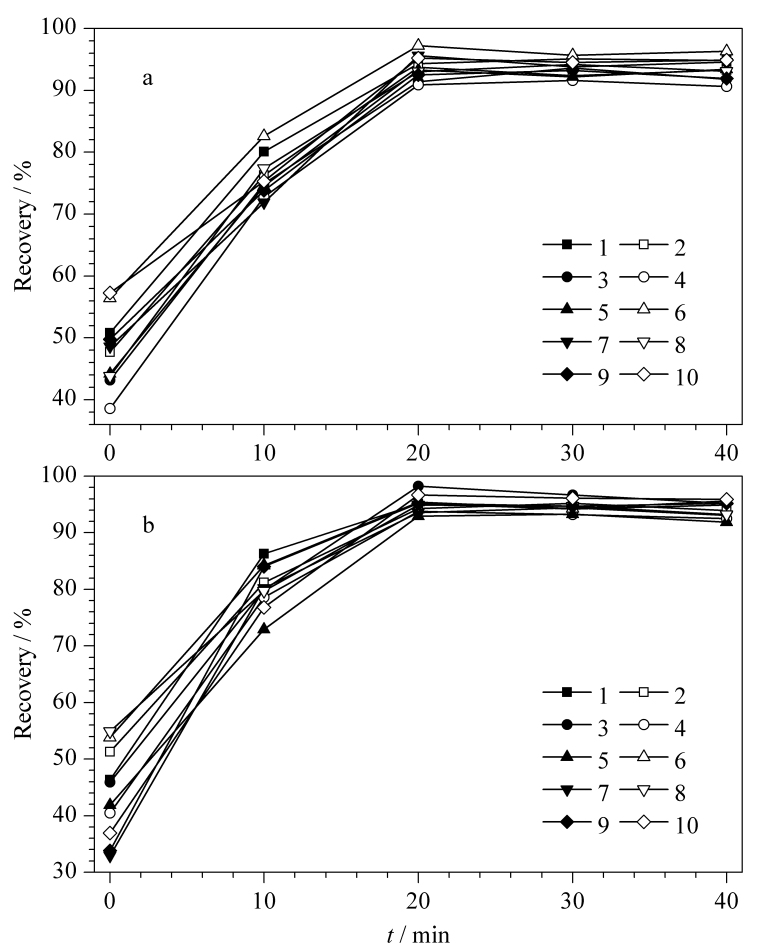
不同超声时间对10种目标分析物提取效率的影响

### 2.5 方法学评价

#### 2.5.1 线性范围、检出限及定量限

将混合标准工作液用乙醇逐级稀释成系列标准工作溶液,按照1.4节中的分析条件进行测定,将各组分的峰面积(*Y*)和对应的质量浓度(*X*, mg/L)进行线性回归,得到相应的线性方程。在阴性样品中分别添加不同水平的待测组分,根据3倍和10倍信噪比(*S/N*)分别确定10种季铵盐类杀菌剂的检出限(LOD, *S/N*=3)和定量限(LOQ, *S/N*=10),结果见表1。十八烷基二甲基苄基氯化铵和二十二烷基三甲基氯化铵在10~200 mg/L范围内线性关系良好,其他目标物在5~100 mg/L范围内线性关系良好,10种目标化合物的相关系数(*R*^2^)均不小于0.9992,检出限为1.42~3.31 mg/L,定量限为4.25~9.94 mg/L,均能满足《化妆品安全技术规范》(2015版)和欧盟化妆品规程的限量要求。

**表1:** 10种目标分析物的线性范围、回归方程、相关系数、检出限和定量限

Compound	Linear range/ (mg/L)	Regression equation	R^2^	LOD/ (mg/L)	LOQ/ (mg/L)
DTAC	5-100	Y=4.262X-2.017	0.9998	1.61	4.82
DDBAC		Y=4.995X-1.422	0.9992	1.46	4.39
BZC		Y=4.614X-2.336	0.9993	1.54	4.62
TTAC		Y=5.004X+12.089	0.9992	1.58	4.73
TDBAC		Y=4.996X+1.862	0.9995	1.45	4.36
HTAC		Y=4.773X-6.145	0.9993	1.63	4.90
HDBAC		Y=6.809X-3.733	0.9997	1.42	4.25
STAC		Y=4.185X-6.243	0.9999	1.64	4.93
SDBAC	10-200	Y=2.200X-5.078	0.9993	3.29	9.87
DocTAC		Y=2.293X+0.304	0.9994	3.31	9.94

*Y*: peak area; *X*: mass concentration, mg/L.

#### 2.5.2 回收率和精密度

分别对3种代表性的阴性样品牙膏(膏体)、漱口水(液体)和洁牙粉(粉末)采用加标法进行回收率和精密度试验。作3个添加水平,每个添加水平作6次平行,精密度和准确度结果见表2, 10种季铵盐类杀菌剂混合标准溶液、阴性样品及阴性加标样品的叠加色谱图见图4。结果表明,在3个不同添加水平下,牙膏、漱口水和洁牙粉中10种季铵盐类杀菌剂的平均回收率为87.9%~103.1%, RSD值均不大于5.5%。

**表2:** 阴性样品中10种目标分析物的加标回收率和相对标准偏差(*n*=6)

Compound	Added/(mg/kg)	Toothpaste			Mouthwash			Dentifrice powder		
		Recovery/%	RSD/%		Recovery/%	RSD/%		Recovery/%		RSD/%
DTAC	50	92.3	4.9		95.2	3.7		94.3	4.5	
	200	93.7	3.2		97.1	3.3		95.1	4.2	
	500	91.9	3.6		96.2	3.5		93.2	3.9	
DDBAC	50	92.3	4.9		94.4	4.2		93.7	4.8	
	200	93.0	4.4		93.2	4.5		94.0	4.8	
	500	100.4	4.3		97.5	3.8		96.8	4.2	
BZC	50	98.2	4.8		98.8	3.6		97.7	4.3	
	200	96.3	3.5		101.4	3.9		97.9	4.5	
	500	99.1	4.2		103.1	3.5		98.3	4.0	
TTAC	50	90.9	4.0		97.9	4.6		92.2	4.4	
	200	95.2	3.9		95.8	4.6		94.8	4.2	
	500	94.9	4.7		96.7	4.2		95.1	4.7	
TDBAC	50	94.1	4.3		102.3	3.6		97.3	3.9	
	200	93.9	3.9		99.4	3.2		97.5	4.1	
	500	98.2	4.5		100.5	3.8		95.2	3.3	
HTAC	50	87.9	4.9		98.9	4.4		91.9	4.9	
	200	90.6	3.3		97.3	3.2		93.0	4.3	
	500	94.8	3.9		99.9	3.7		93.7	4.6	
HDBAC	50	92.7	4.0		98.2	4.0		94.5	4.8	
	200	95.1	4.2		97.3	3.5		93.9	4.0	
	500	94.4	3.8		100.1	4.2		98.1	4.2	
STAC	50	90.9	4.4		95.6	4.7		93.7	4.6	
	200	92.6	3.5		97.3	4.1		94.4	3.6	
	500	99.2	3.9		96.1	4.5		98.0	4.0	
SDBAC	100	90.3	5.1		94.3	4.1		92.2	4.3	
	500	92.2	4.9		96.0	3.4		95.2	4.5	
	1000	92.7	4.5		96.4	3.2		95.6	3.8	
DocTAC	100	90.9	5.5		95.2	4.5		94.9	5.3	
	500	91.8	4.8		93.8	4.3		94.2	4.9	
	1000	96.2	5.0		94.7	3.6		95.8	5.1	

**图4:**
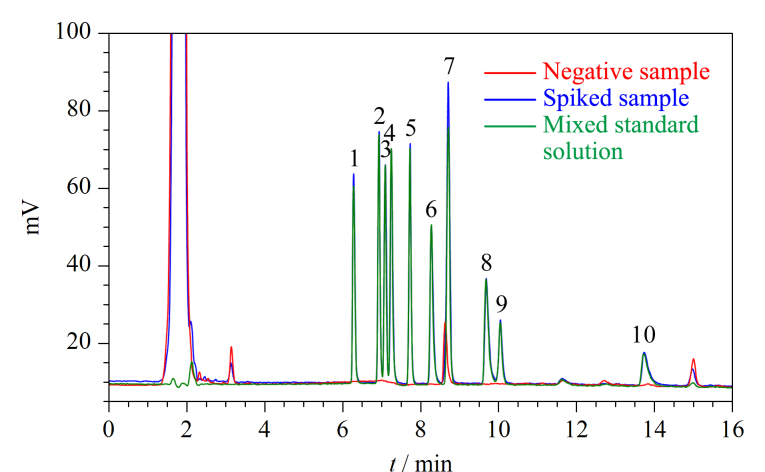
10种目标分析物混合标准溶液、阴性漱口水及阴性漱口水加标样品的色谱图

### 2.6 实际样品检测

应用本研究建立的方法对市售的多品牌多产区的33个漱口水、47个牙膏和29个洁牙粉等常用口腔护理产品进行测定。结果表明,十八烷基二甲基苄基氯化铵和十六烷基二甲基苄基氯化铵检出率相对较高,分别为9.2%和8.3%。大部分产品中的添加量满足法规要求,合格产品的十六烷基二甲基苄基氯化铵的测定值为128~983 mg/kg,十八烷基二甲基苄基氯化铵的测定值为93~774 mg/kg。但有1个牙膏样品中十八烷基二甲基苄基氯化铵的测定值为1511 mg/kg,超出法规中0.1%的限量要求。另外需要特别关注的是,该化合物并未标识在产品配料表中,违反了标签通则的相关规定。

## 3 结论

本研究提出了采用高效液相色谱-蒸发光散射检测器联用同时测定口腔护理产品中10种季铵盐类杀菌剂的方法。本研究使用蒸发光散射检测器将可测定的季铵盐类杀菌剂种类增加到10种。高效液相色谱仪进行色谱分离时采用特定的表面活性剂柱,并对流动相组成情况进行优化,从而实现口腔护理产品中10种季铵盐类杀菌剂的有效分离,最终采用外标法定量,是一种快速有效的定性定量测定方法。本方法的提出可满足化妆品技术规范和欧盟化妆品规程的监管要求,可为口腔护理产品的进出口监管提供技术支持。通过本研究的实际应用发现,口腔护理产品中十八烷基二甲基苄基氯化铵和十六烷基二甲基苄基氯化铵检出率较高,基于环境因素和可能通过食物链在人体中富集的考虑,建议口腔护理产品的生产企业改进生产工艺和配方,尽量减少这两种季铵类盐杀菌剂的添加。
